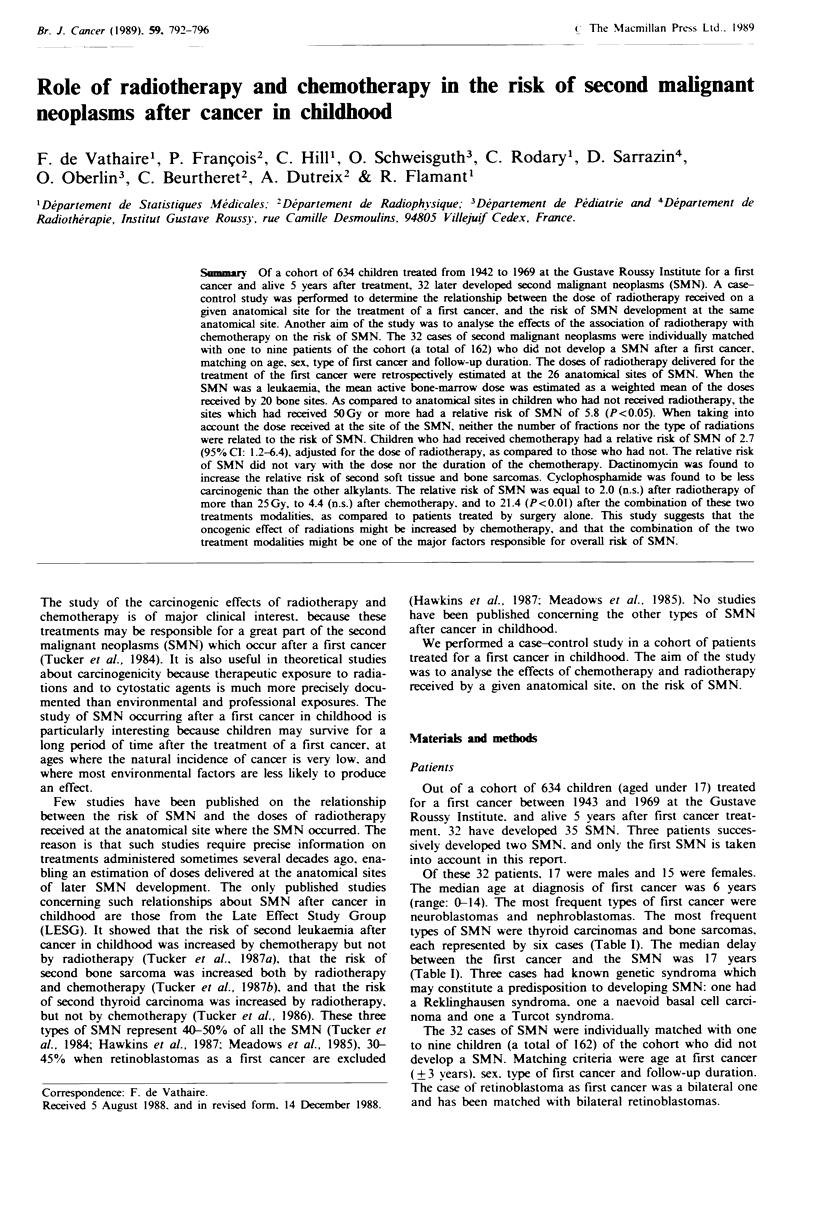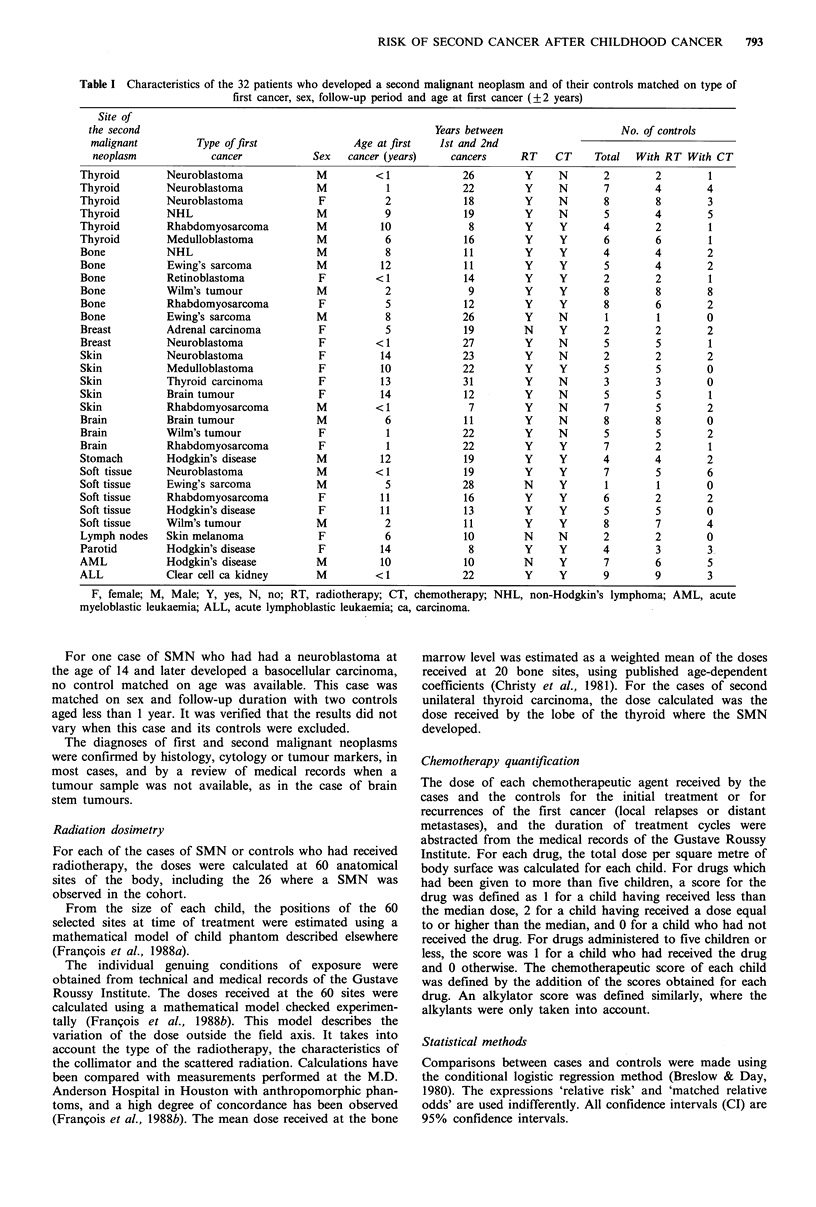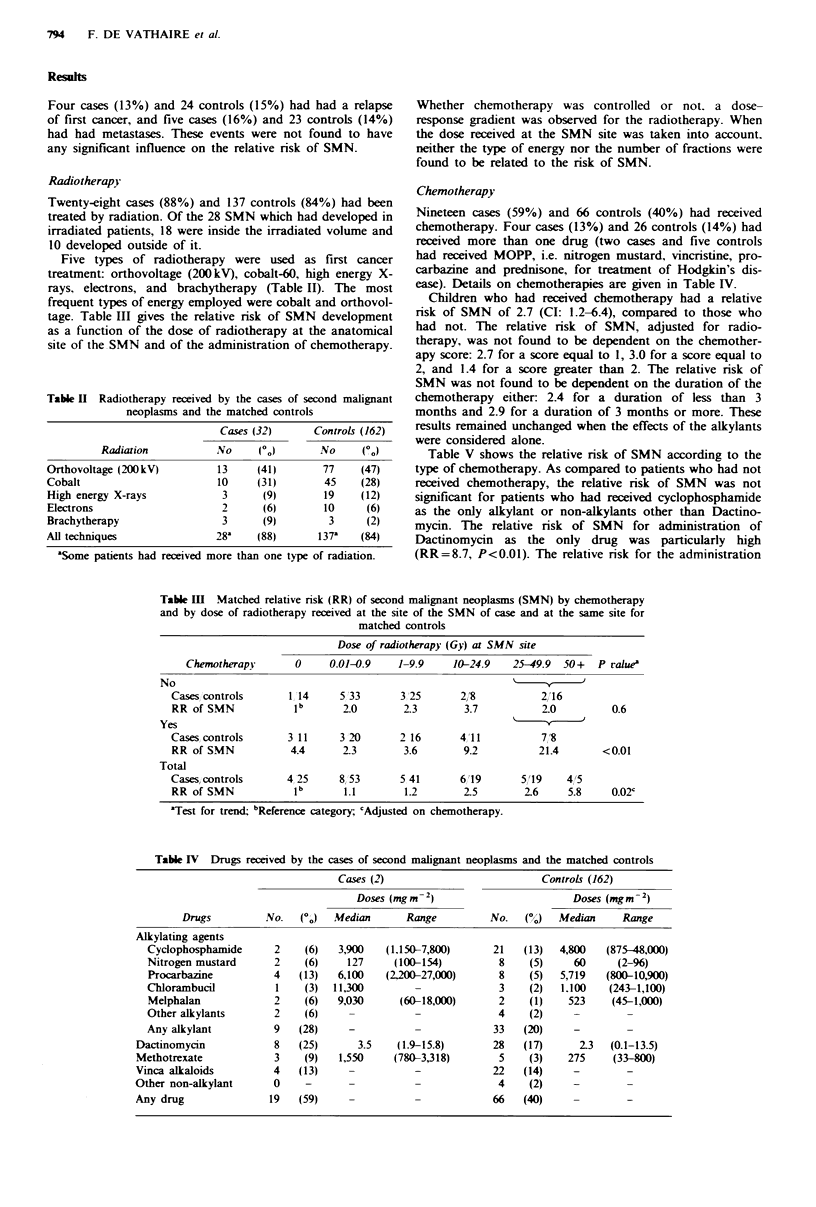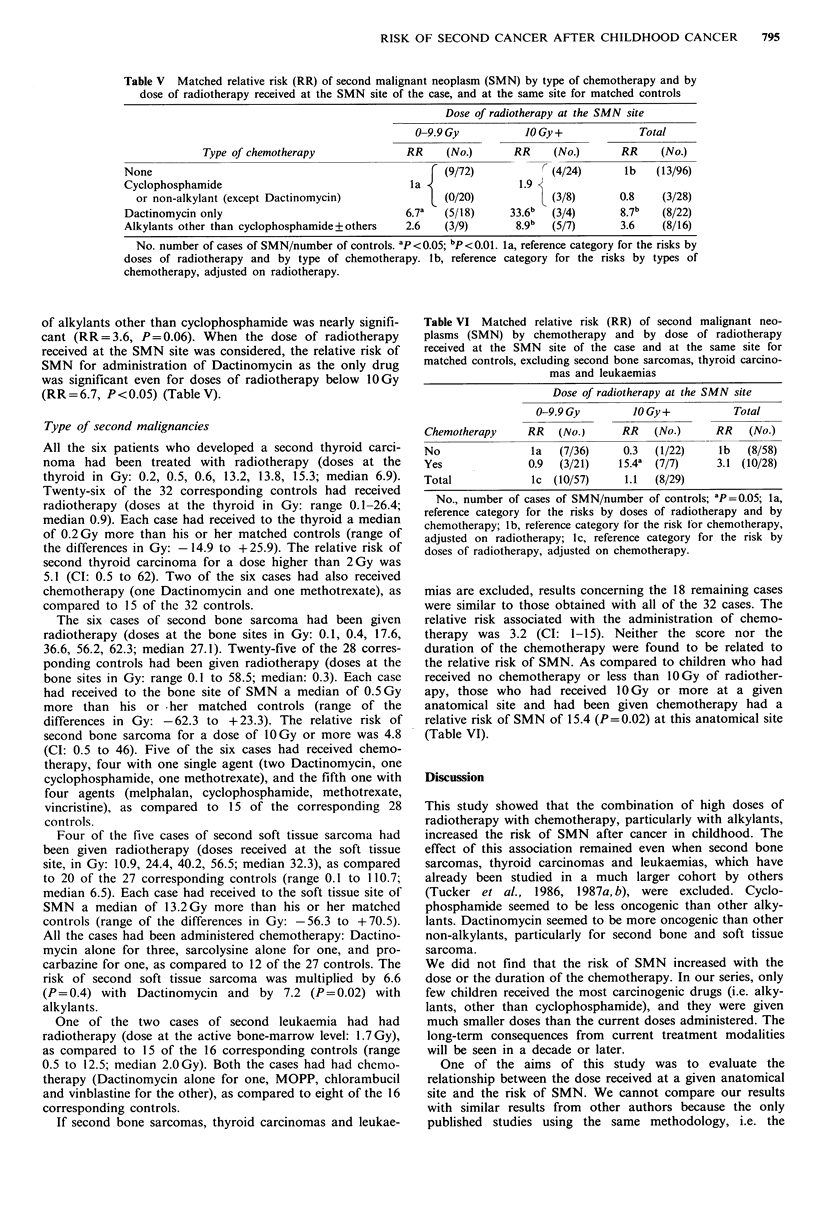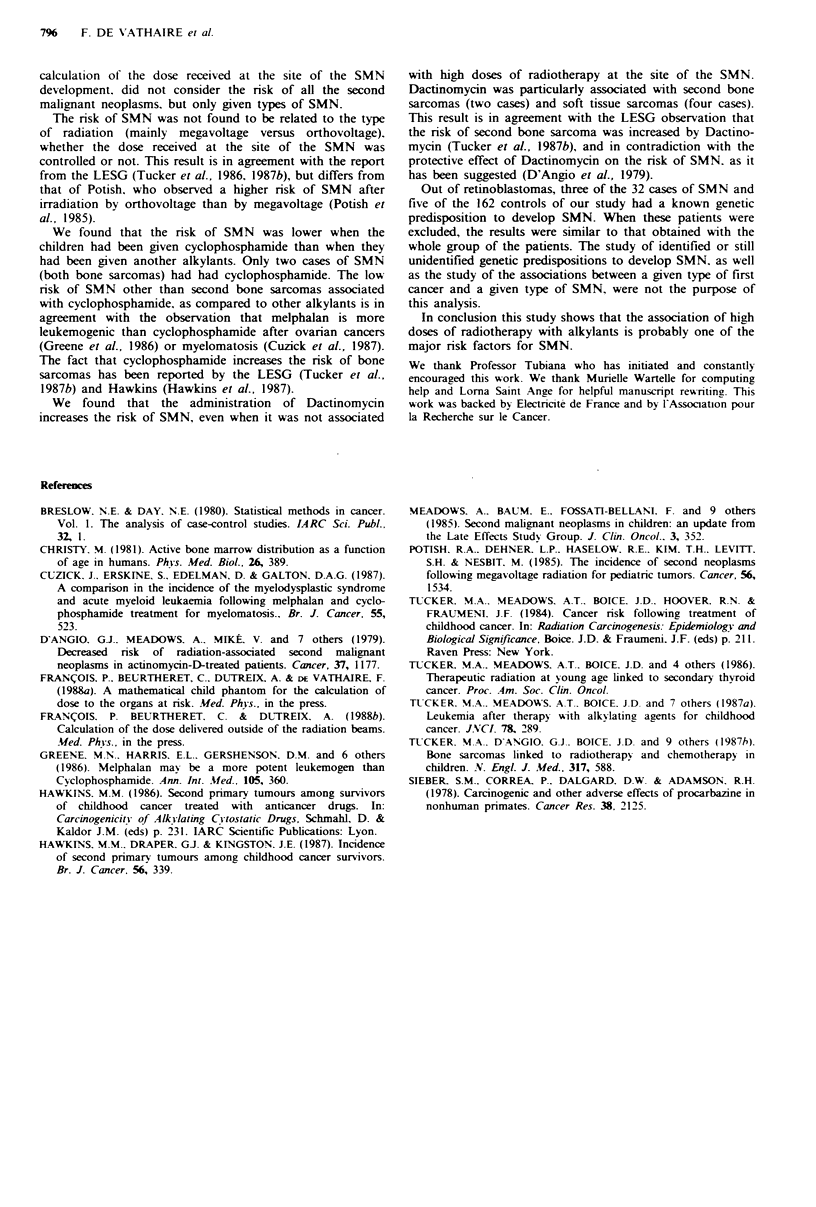# Role of radiotherapy and chemotherapy in the risk of second malignant neoplasms after cancer in childhood.

**DOI:** 10.1038/bjc.1989.165

**Published:** 1989-05

**Authors:** F. de Vathaire, P. FranÃ§ois, C. Hill, O. Schweisguth, C. Rodary, D. Sarrazin, O. Oberlin, C. Beurtheret, A. Dutreix, R. Flamant

**Affiliations:** DÃ©partement de Statistiques MÃ©dicales, Institut Gustave Roussy, Villejuf, France.

## Abstract

Of a cohort of 634 children treated from 1942 to 1969 at the Gustave Roussy Institute for a first cancer and alive 5 years after treatment, 32 later developed second malignant neoplasms (SMN). A case-control study was performed to determine the relationship between the dose of radiotherapy received on a given anatomical site for the treatment of a first cancer, and the risk of SMN development at the same anatomical site. Another aim of the study was to analyse the effects of the association of radiotherapy with chemotherapy on the risk of SMN. The 32 cases of second malignant neoplasms were individually matched with one to nine patients of the cohort (a total of 162) who did not develop a SMN after a first cancer, matching on age, sex, type of first cancer and follow-up duration. The doses of radiotherapy delivered for the treatment of the first cancer were retrospectively estimated at the 26 anatomical sites of SMN. When the SMN was a leukaemia, the mean active bone-marrow dose was estimated as a weighted mean of the doses received by 20 bone sites. As compared to anatomical sites in children who had not received radiotherapy, the sites which had received 50 Gy or more had a relative risk of SMN of 5.8 (P less than 0.05). When taking into account the dose received at the site of the SMN, neither the number of fractions nor the type of radiations were related to the risk of SMN. Children who had received chemotherapy had a relative risk of SMN of 2.7 (95% CI: 1.2-6.4), adjusted for the dose of radiotherapy, as compared to those who had not. The relative risk of SMN did not vary with the dose nor the duration of the chemotherapy. Dactinomycin was found to increase the relative risk of second soft tissue and bone sarcomas. Cyclophosphamide was found to be less carcinogenic than the other alkylants. The relative risk of SMN was equal to 2.0 (n.s.) after radiotherapy of more than 25 Gy, to 4.4 (n.s.) after chemotherapy, and to 21.4 (P less than 0.01) after the combination of these two treatments modalities, as compared to patients treated by surgery alone. This study suggests that the oncogenic effect of radiations might be increased by chemotherapy, and that the combination of the two treatment modalities might be one of the major factors responsible for overall risk of SMN.


Br. J. Cancer (1989), 59, 792-796 © The Macmillan Press Ltd., 1989

### Role of radiotherapy and chemotherapy in the risk of second malignant neoplasms after cancer in childhood

F. de Vathaire[1], P. François[2], C. Hill[1], O. Schweisguth[3], C. Rodary[1], D. Sarrazin[4], O. Oberlin[3], C. Beurtheret[2], A. Dutreix[2] & R. Flamant[1]

[1]*Département de Statistiques Médicales;* [2]*Département de Radiophysique;* [3]*Département de Pédiatrie and* [4]*Département de Radiothérapie, Institut Gustave Roussy, rue Camille Desmoulins, 94805 Villejuif Cedex, France.*

**Summary** Of a cohort of 634 children treated from 1942 to 1969 at the Gustave Roussy Institute for a first cancer and alive 5 years after treatment, 32 later developed second malignant neoplasms (SMN). A case-control study was performed to determine the relationship between the dose of radiotherapy received on a given anatomical site for the treatment of a first cancer, and the risk of SMN development at the same anatomical site. Another aim of the study was to analyse the effects of the association of radiotherapy with chemotherapy on the risk of SMN. The 32 cases of second malignant neoplasms were individually matched with one to nine patients of the cohort (a total of 162) who did not develop a SMN after a first cancer, matching on age, sex, type of first cancer and follow-up duration. The doses of radiotherapy delivered for the treatment of the first cancer were retrospectively estimated at the 26 anatomical sites of SMN. When the SMN was a leukaemia, the mean active bone-marrow dose was estimated as a weighted mean of the doses received by 20 bone sites. As compared to anatomical sites in children who had not received radiotherapy, the sites which had received 50 Gy or more had a relative risk of SMN of 5.8 ($P<0.05$). When taking into account the dose received at the site of the SMN, neither the number of fractions nor the type of radiations were related to the risk of SMN. Children who had received chemotherapy had a relative risk of SMN of 2.7 (95% CI: 1.2–6.4), adjusted for the dose of radiotherapy, as compared to those who had not. The relative risk of SMN did not vary with the dose nor the duration of the chemotherapy. Dactinomycin was found to increase the relative risk of second soft tissue and bone sarcomas. Cyclophosphamide was found to be less carcinogenic than the other alkylants. The relative risk of SMN was equal to 2.0 (n.s.) after radiotherapy of more than 25 Gy, to 4.4 (n.s.) after chemotherapy, and to 21.4 ($P<0.01$) after the combination of these two treatments modalities, as compared to patients treated by surgery alone. This study suggests that the oncogenic effect of radiations might be increased by chemotherapy, and that the combination of the two treatment modalities might be one of the major factors responsible for overall risk of SMN.

The study of the carcinogenic effects of radiotherapy and chemotherapy is of major clinical interest, because these treatments may be responsible for a great part of the second malignant neoplasms (SMN) which occur after a first cancer (Tucker *et al.*, 1984). It is also useful in theoretical studies about carcinogenicity because therapeutic exposure to radiations and to cytostatic agents is much more precisely documented than environmental and professional exposures. The study of SMN occurring after a first cancer in childhood is particularly interesting because children may survive for a long period of time after the treatment of a first cancer, at ages where the natural incidence of cancer is very low, and where most environmental factors are less likely to produce an effect.

Few studies have been published on the relationship between the risk of SMN and the doses of radiotherapy received at the anatomical site where the SMN occurred. The reason is that such studies require precise information on treatments administered sometimes several decades ago, enabling an estimation of doses delivered at the anatomical sites of later SMN development. The only published studies concerning such relationships about SMN after cancer in childhood are those from the Late Effect Study Group (LESG). It showed that the risk of second leukaemia after cancer in childhood was increased by chemotherapy but not by radiotherapy (Tucker *et al.*, 1987*a*), that the risk of second bone sarcoma was increased both by radiotherapy and chemotherapy (Tucker *et al.*, 1987*b*), and that the risk of second thyroid carcinoma was increased by radiotherapy, but not by chemotherapy (Tucker *et al.*, 1986). These three types of SMN represent 40–50% of all the SMN (Tucker *et al.*, 1984; Hawkins *et al.*, 1987; Meadows *et al.*, 1985), 30–45% when retinoblastomas as a first cancer are excluded (Hawkins *et al.*, 1987; Meadows *et al.*, 1985). No studies have been published concerning the other types of SMN after cancer in childhood.

We performed a case-control study in a cohort of patients treated for a first cancer in childhood. The aim of the study was to analyse the effects of chemotherapy and radiotherapy received by a given anatomical site, on the risk of SMN.

### Materials and methods

### *Patients*

Out of a cohort of 634 children (aged under 17) treated for a first cancer between 1943 and 1969 at the Gustave Roussy Institute, and alive 5 years after first cancer treatment, 32 have developed 35 SMN. Three patients successively developed two SMN, and only the first SMN is taken into account in this report.

Of these 32 patients, 17 were males and 15 were females. The median age at diagnosis of first cancer was 6 years (range: 0–14). The most frequent types of first cancer were neuroblastomas and nephroblastomas. The most frequent types of SMN were thyroid carcinomas and bone sarcomas, each represented by six cases (Table I). The median delay between the first cancer and the SMN was 17 years (Table I). Three cases had known genetic syndroma which may constitute a predisposition to developing SMN: one had a Reklinghausen syndroma, one a naevoid basal cell carcinoma and one a Turcot syndroma.

The 32 cases of SMN were individually matched with one to nine children (a total of 162) of the cohort who did not develop a SMN. Matching criteria were age at first cancer (±3 years), sex, type of first cancer and follow-up duration. The case of retinoblastoma as first cancer was a bilateral one and has been matched with bilateral retinoblastomas.

Correspondence: F. de Vathaire.
Received 5 August 1988, and in revised form, 14 December 1988.

**Table I** Characteristics of the 32 patients who developed a second malignant neoplasm and of their controls matched on type of first cancer, sex, follow-up period and age at first cancer (±2 years)

| *Site of the second malignant neoplasm* | *Type of first cancer* | *Sex* | *Age at first cancer (years)* | *Years between 1st and 2nd cancers* | *RT* | *CT* | *No. of controls* | | |
|---|---|---|---|---|---|---|---|---|---|
| | | | | | | | *Total* | *With RT* | *With CT* |
| Thyroid | Neuroblastoma | M | <1 | 26 | Y | N | 2 | 2 | 1 |
| Thyroid | Neuroblastoma | M | 1 | 22 | Y | N | 7 | 4 | 4 |
| Thyroid | Neuroblastoma | F | 2 | 18 | Y | N | 8 | 8 | 3 |
| Thyroid | NHL | M | 9 | 19 | Y | N | 5 | 4 | 5 |
| Thyroid | Rhabdomyosarcoma | M | 10 | 8 | Y | Y | 4 | 2 | 1 |
| Thyroid | Medulloblastoma | M | 6 | 16 | Y | Y | 6 | 6 | 1 |
| Bone | NHL | M | 8 | 11 | Y | Y | 4 | 4 | 2 |
| Bone | Ewing's sarcoma | M | 12 | 11 | Y | Y | 5 | 4 | 2 |
| Bone | Retinoblastoma | F | <1 | 14 | Y | Y | 2 | 2 | 1 |
| Bone | Wilm's tumour | M | 2 | 9 | Y | Y | 8 | 8 | 8 |
| Bone | Rhabdomyosarcoma | F | 5 | 12 | Y | Y | 8 | 6 | 2 |
| Bone | Ewing's sarcoma | M | 8 | 26 | Y | N | 1 | 1 | 0 |
| Breast | Adrenal carcinoma | F | 5 | 19 | N | Y | 2 | 2 | 2 |
| Breast | Neuroblastoma | F | <1 | 27 | Y | N | 5 | 5 | 1 |
| Skin | Neuroblastoma | F | 14 | 23 | Y | N | 2 | 2 | 2 |
| Skin | Medulloblastoma | F | 10 | 22 | Y | Y | 5 | 5 | 0 |
| Skin | Thyroid carcinoma | F | 13 | 31 | Y | N | 3 | 3 | 0 |
| Skin | Brain tumour | F | 14 | 12 | Y | N | 5 | 5 | 1 |
| Skin | Rhabdomyosarcoma | M | <1 | 7 | Y | N | 7 | 5 | 2 |
| Brain | Brain tumour | M | 6 | 11 | Y | N | 8 | 8 | 0 |
| Brain | Wilm's tumour | F | 1 | 22 | Y | N | 5 | 5 | 2 |
| Brain | Rhabdomyosarcoma | F | 1 | 22 | Y | Y | 7 | 2 | 1 |
| Stomach | Hodgkin's disease | M | 12 | 19 | Y | Y | 4 | 4 | 2 |
| Soft tissue | Neuroblastoma | M | <1 | 19 | Y | Y | 7 | 5 | 6 |
| Soft tissue | Ewing's sarcoma | M | 5 | 28 | N | Y | 1 | 1 | 0 |
| Soft tissue | Rhabdomyosarcoma | F | 11 | 16 | Y | Y | 6 | 2 | 2 |
| Soft tissue | Hodgkin's disease | F | 11 | 13 | Y | Y | 5 | 5 | 0 |
| Soft tissue | Wilm's tumour | M | 2 | 11 | Y | Y | 8 | 7 | 4 |
| Lymph nodes | Skin melanoma | F | 6 | 10 | N | N | 2 | 2 | 0 |
| Parotid | Hodgkin's disease | F | 14 | 8 | Y | Y | 4 | 3 | 3 |
| AML | Hodgkin's disease | M | 10 | 10 | N | Y | 7 | 6 | 5 |
| ALL | Clear cell ca kidney | M | <1 | 22 | Y | Y | 9 | 9 | 3 |

F, female; M, Male; Y, yes, N, no; RT, radiotherapy; CT, chemotherapy; NHL, non-Hodgkin's lymphoma; AML, acute myeloblastic leukaemia; ALL, acute lymphoblastic leukaemia; ca, carcinoma.

For one case of SMN who had had a neuroblastoma at the age of 14 and later developed a basocellular carcinoma, no control matched on age was available. This case was matched on sex and follow-up duration with two controls aged less than 1 year. It was verified that the results did not vary when this case and its controls were excluded.

The diagnoses of first and second malignant neoplasms were confirmed by histology, cytology or tumour markers, in most cases, and by a review of medical records when a tumour sample was not available, as in the case of brain stem tumours.

### *Radiation dosimetry*

For each of the cases of SMN or controls who had received radiotherapy, the doses were calculated at 60 anatomical sites of the body, including the 26 where a SMN was observed in the cohort.

From the size of each child, the positions of the 60 selected sites at time of treatment were estimated using a mathematical model of child phantom described elsewhere (François *et al.*, 1988*a*).

The individual genuing conditions of exposure were obtained from technical and medical records of the Gustave Roussy Institute. The doses received at the 60 sites were calculated using a mathematical model checked experimentally (François *et al.*, 1988*b*). This model describes the variation of the dose outside the field axis. It takes into account the type of the radiotherapy, the characteristics of the collimator and the scattered radiation. Calculations have been compared with measurements performed at the M.D. Anderson Hospital in Houston with anthropomorphic phantoms, and a high degree of concordance has been observed (François *et al.*, 1988*b*). The mean dose received at the bone marrow level was estimated as a weighted mean of the doses received at 20 bone sites, using published age-dependent coefficients (Christy *et al.*, 1981). For the cases of second unilateral thyroid carcinoma, the dose calculated was the dose received by the lobe of the thyroid where the SMN developed.

### *Chemotherapy quantification*

The dose of each chemotherapeutic agent received by the cases and the controls for the initial treatment or for recurrences of the first cancer (local relapses or distant metastases), and the duration of treatment cycles were abstracted from the medical records of the Gustave Roussy Institute. For each drug, the total dose per square metre of body surface was calculated for each child. For drugs which had been given to more than five children, a score for the drug was defined as 1 for a child having received less than the median dose, 2 for a child having received a dose equal to or higher than the median, and 0 for a child who had not received the drug. For drugs administered to five children or less, the score was 1 for a child who had received the drug and 0 otherwise. The chemotherapeutic score of each child was defined by the addition of the scores obtained for each drug. An alkylator score was defined similarly, where the alkylants were only taken into account.

### *Statistical methods*

Comparisons between cases and controls were made using the conditional logistic regression method (Breslow & Day, 1980). The expressions 'relative risk' and 'matched relative odds' are used indifferently. All confidence intervals (CI) are 95% confidence intervals.

### Results

Four cases (13%) and 24 controls (15%) had had a relapse of first cancer, and five cases (16%) and 23 controls (14%) had had metastases. These events were not found to have any significant influence on the relative risk of SMN.

### *Radiotherapy*

Twenty-eight cases (88%) and 137 controls (84%) had been treated by radiation. Of the 28 SMN which had developed in irradiated patients, 18 were inside the irradiated volume and 10 developed outside of it.

Five types of radiotherapy were used as first cancer treatment: orthovoltage (200 kV), cobalt-60, high energy X-rays, electrons, and brachytherapy (Table II). The most frequent types of energy employed were cobalt and orthovoltage. Table III gives the relative risk of SMN development as a function of the dose of radiotherapy at the anatomical site of the SMN and of the administration of chemotherapy.

**Table II** Radiotherapy received by the cases of second malignant neoplasms and the matched controls

| *Radiation* | *Cases (32)* *No* | (%) | *Controls (162)* *No* | (%) |
|---|---|---|---|---|
| Orthovoltage (200 kV) | 13 | (41) | 77 | (47) |
| Cobalt | 10 | (31) | 45 | (28) |
| High energy X-rays | 3 | (9) | 19 | (12) |
| Electrons | 2 | (6) | 10 | (6) |
| Brachytherapy | 3 | (9) | 3 | (2) |
| All techniques | 28[a] | (88) | 137[a] | (84) |

[a]Some patients had received more than one type of radiation.

Whether chemotherapy was controlled or not, a dose–response gradient was observed for the radiotherapy. When the dose received at the SMN site was taken into account, neither the type of energy nor the number of fractions were found to be related to the risk of SMN.

### *Chemotherapy*

Nineteen cases (59%) and 66 controls (40%) had received chemotherapy. Four cases (13%) and 26 controls (14%) had received more than one drug (two cases and five controls had received MOPP, i.e. nitrogen mustard, vincristine, procarbazine and prednisone, for treatment of Hodgkin's disease). Details on chemotherapies are given in Table IV.

Children who had received chemotherapy had a relative risk of SMN of 2.7 (CI: 1.2–6.4), compared to those who had not. The relative risk of SMN, adjusted for radiotherapy, was not found to be dependent on the chemotherapy score: 2.7 for a score equal to 1, 3.0 for a score equal to 2, and 1.4 for a score greater than 2. The relative risk of SMN was not found to be dependent on the duration of the chemotherapy either: 2.4 for a duration of less than 3 months and 2.9 for a duration of 3 months or more. These results remained unchanged when the effects of the alkylants were considered alone.

Table V shows the relative risk of SMN according to the type of chemotherapy. As compared to patients who had not received chemotherapy, the relative risk of SMN was not significant for patients who had received cyclophosphamide as the only alkylant or non-alkylants other than Dactinomycin. The relative risk of SMN for administration of Dactinomycin as the only drug was particularly high (RR = 8.7, $P < 0.01$). The relative risk for the administration

**Table III** Matched relative risk (RR) of second malignant neoplasms (SMN) by chemotherapy and by dose of radiotherapy received at the site of the SMN of case and at the same site for matched controls

| | *Dose of radiotherapy (Gy) at SMN site* | | | | | | |
|---|---|---|---|---|---|---|---|
| *Chemotherapy* | *0* | *0.01–0.9* | *1–9.9* | *10–24.9* | *25–49.9* | *50+* | *P value*[a] |
| No | | | | | | | |
| Cases/controls | 1/14 | 5/33 | 3/25 | 2/8 | 2/16 (25–49.9 and 50+ combined) | | |
| RR of SMN | 1[b] | 2.0 | 2.3 | 3.7 | 2.0 (25–49.9 and 50+ combined) | | 0.6 |
| Yes | | | | | | | |
| Cases/controls | 3/11 | 3/20 | 2/16 | 4/11 | 7/8 (25–49.9 and 50+ combined) | | |
| RR of SMN | 4.4 | 2.3 | 3.6 | 9.2 | 21.4 (25–49.9 and 50+ combined) | | <0.01 |
| Total | | | | | | | |
| Cases/controls | 4/25 | 8/53 | 5/41 | 6/19 | 5/19 | 4/5 | |
| RR of SMN | 1[b] | 1.1 | 1.2 | 2.5 | 2.6 | 5.8 | 0.02[c] |

[a]Test for trend; [b]Reference category; [c]Adjusted on chemotherapy.

**Table IV** Drugs received by the cases of second malignant neoplasms and the matched controls

| | *Cases (2)* | | | | *Controls (162)* | | | |
|---|---|---|---|---|---|---|---|---|
| | | | *Doses (mg m$^{-2}$)* | | | | *Doses (mg m$^{-2}$)* | |
| *Drugs* | *No.* | (%) | *Median* | *Range* | *No.* | (%) | *Median* | *Range* |
| Alkylating agents | | | | | | | | |
| Cyclophosphamide | 2 | (6) | 3,900 | (1,150–7,800) | 21 | (13) | 4,800 | (875–48,000) |
| Nitrogen mustard | 2 | (6) | 127 | (100–154) | 8 | (5) | 60 | (2–96) |
| Procarbazine | 4 | (13) | 6,100 | (2,200–27,000) | 8 | (5) | 5,719 | (800–10,900) |
| Chlorambucil | 1 | (3) | 11,300 | – | 3 | (2) | 1,100 | (243–1,100) |
| Melphalan | 2 | (6) | 9,030 | (60–18,000) | 2 | (1) | 523 | (45–1,000) |
| Other alkylants | 2 | (6) | – | – | 4 | (2) | – | – |
| Any alkylant | 9 | (28) | – | – | 33 | (20) | – | – |
| Dactinomycin | 8 | (25) | 3.5 | (1.9–15.8) | 28 | (17) | 2.3 | (0.1–13.5) |
| Methotrexate | 3 | (9) | 1,550 | (780–3,318) | 5 | (3) | 275 | (33–800) |
| Vinca alkaloids | 4 | (13) | – | – | 22 | (14) | – | – |
| Other non-alkylant | 0 | – | – | – | 4 | (2) | – | – |
| Any drug | 19 | (59) | – | – | 66 | (40) | – | – |

**Table V** Matched relative risk (RR) of second malignant neoplasm (SMN) by type of chemotherapy and by dose of radiotherapy received at the SMN site of the case, and at the same site for matched controls

| | *Dose of radiotherapy at the SMN site* | | | | | |
|---|---|---|---|---|---|---|
| | *0–9.9 Gy* | | *10 Gy+* | | *Total* | |
| *Type of chemotherapy* | *RR* | *(No.)* | *RR* | *(No.)* | *RR* | *(No.)* |
| None | 1a | (9/72) | 1.9 | (4/24) | 1b | (13/96) |
| Cyclophosphamide or non-alkylant (except Dactinomycin) | | (0/20) | | (3/8) | 0.8 | (3/28) |
| Dactinomycin only | 6.7[a] | (5/18) | 33.6[b] | (3/4) | 8.7[b] | (8/22) |
| Alkylants other than cyclophosphamide ± others | 2.6 | (3/9) | 8.9[b] | (5/7) | 3.6 | (8/16) |

No. number of cases of SMN/number of controls. [a]$P<0.05$; [b]$P<0.01$. 1a, reference category for the risks by doses of radiotherapy and by type of chemotherapy. 1b, reference category for the risks by types of chemotherapy, adjusted on radiotherapy.

of alkylants other than cyclophosphamide was nearly significant (RR = 3.6, $P=0.06$). When the dose of radiotherapy received at the SMN site was considered, the relative risk of SMN for administration of Dactinomycin as the only drug was significant even for doses of radiotherapy below 10 Gy (RR = 6.7, $P<0.05$) (Table V).

*Type of second malignancies*

All the six patients who developed a second thyroid carcinoma had been treated with radiotherapy (doses at the thyroid in Gy: 0.2, 0.5, 0.6, 13.2, 13.8, 15.3; median 6.9). Twenty-six of the 32 corresponding controls had received radiotherapy (doses at the thyroid in Gy: range 0.1–26.4; median 0.9). Each case had received to the thyroid a median of 0.2 Gy more than his or her matched controls (range of the differences in Gy: −14.9 to +25.9). The relative risk of second thyroid carcinoma for a dose higher than 2 Gy was 5.1 (CI: 0.5 to 62). Two of the six cases had also received chemotherapy (one Dactinomycin and one methotrexate), as compared to 15 of the 32 controls.

The six cases of second bone sarcoma had been given radiotherapy (doses at the bone sites in Gy: 0.1, 0.4, 17.6, 36.6, 56.2, 62.3; median 27.1). Twenty-five of the 28 corresponding controls had been given radiotherapy (doses at the bone sites in Gy: range 0.1 to 58.5; median: 0.3). Each case had received to the bone site of SMN a median of 0.5 Gy more than his or her matched controls (range of the differences in Gy: −62.3 to +23.3). The relative risk of second bone sarcoma for a dose of 10 Gy or more was 4.8 (CI: 0.5 to 46). Five of the six cases had received chemotherapy, four with one single agent (two Dactinomycin, one cyclophosphamide, one methotrexate), and the fifth one with four agents (melphalan, cyclophosphamide, methotrexate, vincristine), as compared to 15 of the corresponding 28 controls.

Four of the five cases of second soft tissue sarcoma had been given radiotherapy (doses received at the soft tissue site, in Gy: 10.9, 24.4, 40.2, 56.5; median 32.3), as compared to 20 of the 27 corresponding controls (range 0.1 to 110.7; median 6.5). Each case had received to the soft tissue site of SMN a median of 13.2 Gy more than his or her matched controls (range of the differences in Gy: −56.3 to +70.5). All the cases had been administered chemotherapy: Dactinomycin alone for three, sarcolysine alone for one, and procarbazine for one, as compared to 12 of the 27 controls. The risk of second soft tissue sarcoma was multiplied by 6.6 ($P=0.4$) with Dactinomycin and by 7.2 ($P=0.02$) with alkylants.

One of the two cases of second leukaemia had had radiotherapy (dose at the active bone-marrow level: 1.7 Gy), as compared to 15 of the 16 corresponding controls (range 0.5 to 12.5; median 2.0 Gy). Both the cases had had chemotherapy (Dactinomycin alone for one, MOPP, chlorambucil and vinblastine for the other), as compared to eight of the 16 corresponding controls.

If second bone sarcomas, thyroid carcinomas and leukaemias are excluded, results concerning the 18 remaining cases were similar to those obtained with all of the 32 cases. The relative risk associated with the administration of chemotherapy was 3.2 (CI: 1–15). Neither the score nor the duration of the chemotherapy were found to be related to the relative risk of SMN. As compared to children who had received no chemotherapy or less than 10 Gy of radiotherapy, those who had received 10 Gy or more at a given anatomical site and had been given chemotherapy had a relative risk of SMN of 15.4 ($P=0.02$) at this anatomical site (Table VI).

**Table VI** Matched relative risk (RR) of second malignant neoplasms (SMN) by chemotherapy and by dose of radiotherapy received at the SMN site of the case and at the same site for matched controls, excluding second bone sarcomas, thyroid carcinomas and leukaemias

| | *Dose of radiotherapy at the SMN site* | | | | | |
|---|---|---|---|---|---|---|
| | *0–9.9 Gy* | | *10 Gy+* | | *Total* | |
| *Chemotherapy* | *RR* | *(No.)* | *RR* | *(No.)* | *RR* | *(No.)* |
| No | 1a | (7/36) | 0.3 | (1/22) | 1b | (8/58) |
| Yes | 0.9 | (3/21) | 15.4[a] | (7/7) | 3.1 | (10/28) |
| Total | 1c | (10/57) | 1.1 | (8/29) | | |

No., number of cases of SMN/number of controls; [a]$P=0.05$; 1a, reference category for the risks by doses of radiotherapy and by chemotherapy; 1b, reference category for the risk for chemotherapy, adjusted on radiotherapy; 1c, reference category for the risk by doses of radiotherapy, adjusted on chemotherapy.

### Discussion

This study showed that the combination of high doses of radiotherapy with chemotherapy, particularly with alkylants, increased the risk of SMN after cancer in childhood. The effect of this association remained even when second bone sarcomas, thyroid carcinomas and leukaemias, which have already been studied in a much larger cohort by others (Tucker *et al.*, 1986, 1987*a*,*b*), were excluded. Cyclophosphamide seemed to be less oncogenic than other alkylants. Dactinomycin seemed to be more oncogenic than other non-alkylants, particularly for second bone and soft tissue sarcoma.

We did not find that the risk of SMN increased with the dose or the duration of the chemotherapy. In our series, only few children received the most carcinogenic drugs (i.e. alkylants, other than cyclophosphamide), and they were given much smaller doses than the current doses administered. The long-term consequences from current treatment modalities will be seen in a decade or later.

One of the aims of this study was to evaluate the relationship between the dose received at a given anatomical site and the risk of SMN. We cannot compare our results with similar results from other authors because the only published studies using the same methodology, i.e. the calculation of the dose received at the site of the SMN development, did not consider the risk of all the second malignant neoplasms, but only given types of SMN.

The risk of SMN was not found to be related to the type of radiation (mainly megavoltage versus orthovoltage), whether the dose received at the site of the SMN was controlled or not. This result is in agreement with the report from the LESG (Tucker *et al.*, 1986, 1987*b*), but differs from that of Potish, who observed a higher risk of SMN after irradiation by orthovoltage than by megavoltage (Potish *et al.*, 1985).

We found that the risk of SMN was lower when the children had been given cyclophosphamide than when they had been given another alkylants. Only two cases of SMN (both bone sarcomas) had had cyclophosphamide. The low risk of SMN other than second bone sarcomas associated with cyclophosphamide, as compared to other alkylants is in agreement with the observation that melphalan is more leukemogenic than cyclophosphamide after ovarian cancers (Greene *et al.*, 1986) or myelomatosis (Cuzick *et al.*, 1987). The fact that cyclophosphamide increases the risk of bone sarcomas has been reported by the LESG (Tucker *et al.*, 1987*b*) and Hawkins (Hawkins *et al.*, 1987).

We found that the administration of Dactinomycin increases the risk of SMN, even when it was not associated with high doses of radiotherapy at the site of the SMN. Dactinomycin was particularly associated with second bone sarcomas (two cases) and soft tissue sarcomas (four cases). This result is in agreement with the LESG observation that the risk of second bone sarcoma was increased by Dactinomycin (Tucker *et al.*, 1987*b*), and in contradiction with the protective effect of Dactinomycin on the risk of SMN, as it has been suggested (D'Angio *et al.*, 1979).

Out of retinoblastomas, three of the 32 cases of SMN and five of the 162 controls of our study had a known genetic predisposition to develop SMN. When these patients were excluded, the results were similar to that obtained with the whole group of the patients. The study of identified or still unidentified genetic predispositions to develop SMN, as well as the study of the associations between a given type of first cancer and a given type of SMN, were not the purpose of this analysis.

In conclusion this study shows that the association of high doses of radiotherapy with alkylants is probably one of the major risk factors for SMN.

We thank Professor Tubiana who has initiated and constantly encouraged this work. We thank Murielle Wartelle for computing help and Lorna Saint Ange for helpful manuscript rewriting. This work was backed by Electricité de France and by l'Association pour la Recherche sur le Cancer.

### References

BRESLOW, N.E. & DAY, N.E. (1980). Statistical methods in cancer. Vol. 1. The analysis of case-control studies. *IARC Sci. Publ.*, **32**, 1.

CHRISTY, M. (1981). Active bone marrow distribution as a function of age in humans. *Phys. Med. Biol.*, **26**, 389.

CUZICK, J., ERSKINE, S., EDELMAN, D. & GALTON, D.A.G. (1987). A comparison in the incidence of the myelodysplastic syndrome and acute myeloid leukaemia following melphalan and cyclophosphamide treatment for myelomatosis., *Br. J. Cancer*, **55**, 523.

D'ANGIO, G.J., MEADOWS, A., MIKÉ, V. and 7 others (1979). Decreased risk of radiation-associated second malignant neoplasms in actinomycin-D-treated patients. *Cancer*, **37**, 1177.

FRANÇOIS, P., BEURTHERET, C., DUTREIX, A. & DE VATHAIRE, F. (1988*a*). A mathematical child phantom for the calculation of dose to the organs at risk. *Med. Phys.*, in the press.

FRANÇOIS, P. BEURTHERET, C. & DUTREIX, A. (1988*b*). Calculation of the dose delivered outside of the radiation beams. *Med. Phys.*, in the press.

GREENE, M.N., HARRIS, E.L., GERSHENSON, D.M. and 6 others (1986). Melphalan may be a more potent leukemogen than Cyclophosphamide. *Ann. Int. Med.*, **105**, 360.

HAWKINS, M.M. (1986). Second primary tumours among survivors of childhood cancer treated with anticancer drugs. In: *Carcinogenicity of Alkylating Cytostatic Drugs*, Schmahl, D. & Kaldor J.M. (eds) p. 231. IARC Scientific Publications: Lyon.

HAWKINS, M.M., DRAPER, G.J. & KINGSTON, J.E. (1987). Incidence of second primary tumours among childhood cancer survivors. *Br. J. Cancer*, **56**, 339.

MEADOWS, A., BAUM, E., FOSSATI-BELLANI, F. and 9 others (1985). Second malignant neoplasms in children: an update from the Late Effects Study Group. *J. Clin. Oncol.*, **3**, 352.

POTISH, R.A., DEHNER, L.P., HASELOW, R.E., KIM, T.H., LEVITT, S.H. & NESBIT, M. (1985). The incidence of second neoplasms following megavoltage radiation for pediatric tumors. *Cancer*, **56**, 1534.

TUCKER, M.A., MEADOWS, A.T., BOICE, J.D., HOOVER, R.N. & FRAUMENI, J.F. (1984). Cancer risk following treatment of childhood cancer. In: *Radiation Carcinogenesis: Epidemiology and Biological Significance*, Boice, J.D. & Fraumeni, J.F. (eds) p. 211. Raven Press: New York.

TUCKER, M.A., MEADOWS, A.T., BOICE, J.D. and 4 others (1986). Therapeutic radiation at young age linked to secondary thyroid cancer. *Proc. Am. Soc. Clin. Oncol.*

TUCKER, M.A., MEADOWS, A.T., BOICE, J.D. and 7 others (1987*a*). Leukemia after therapy with alkylating agents for childhood cancer. *JNCI*, **78**, 289.

TUCKER, M.A., D'ANGIO, G.J., BOICE, J.D. and 9 others (1987*b*). Bone sarcomas linked to radiotherapy and chemotherapy in children. *N. Engl. J. Med.*, **317**, 588.

SIEBER, S.M., CORREA, P., DALGARD, D.W. & ADAMSON, R.H. (1978). Carcinogenic and other adverse effects of procarbazine in nonhuman primates. *Cancer Res.* **38**, 2125.